# Trends in incidence of recorded diagnosis of osteoporosis, osteopenia, and fragility fractures in people aged 50 years and above: retrospective cohort study using UK primary care data

**DOI:** 10.1007/s00198-023-06739-1

**Published:** 2023-05-10

**Authors:** Christina Avgerinou, Irene Petersen, Andrew Clegg, Robert M. West, David Osborn, Kate Walters

**Affiliations:** 1 Department of Primary Care and Population Health, University College London, Royal Free Campus, Rowland Hill Street, London, NW3 2PF UK; 2 Academic Unit for Ageing and Stroke Research, Bradford Institute for Health Research, University of Leeds, Leeds, UK; 3 Leeds Institute of Health Sciences, University of Leeds, Leeds, UK; 4 Division of Psychiatry, University College London, Leeds, UK; 5 Camden and Islington NHS Foundation Trust, London, UK

**Keywords:** Electronic health records, Fragility fracture, Incidence, Older people, Osteopenia, Osteoporosis, Primary care

## Abstract

**Summary:**

This study used primary care data to estimate the incidence of recorded diagnosis of osteoporosis, osteopenia, and fragility fracture in the UK during 2000–2018 accounting for age, sex, calendar year and social deprivation. More than 3 million people aged 50–99 years were included. We found that men living in the most deprived areas had a 45% higher risk of being diagnosed with osteoporosis and 50% higher risk of fragility fracture compared to men living in the least deprived areas.

**Purpose:**

a) To estimate the incidence trends of a recorded diagnosis of osteoporosis, osteopenia, and fragility fracture in the UK over time; b) to describe differences according to age, sex, and social deprivation.

**Methods:**

This is a longitudinal population-based cohort study using routinely collected primary care data obtained via IQVIA Medical Research Database (IMRD). All patients aged 50–99 years registered with a practice participating in THIN (The Health Improvement Network) between 2000–2018 were included. The first recorded diagnosis of osteoporosis, osteopenia, or fragility fracture was used to estimate incidence rates (IR) per 10,000 person-years at risk. Poisson regression was used to provide Incidence Rate Ratios (IRR) adjusted by age, sex, social deprivation, calendar year, and practice effect.

**Results:**

The year-specific adjusted IRR of recorded osteoporosis was highest in 2009 in women [IRR 1.44(95%CI 1.38–1.50)], whereas in men it was highest in 2013–2014 [IRR 1.94(95%CI 1.72–2.18)] compared to 2000. The year-specific adjusted IRR of fragility fracture was highest in 2012 in women [IRR 1.77(95%CI 1.69–1.85)], whereas in men it was highest in 2013 [IRR 1.64(95%CI 1.51–1.78)] compared to 2000. Men in the most deprived areas had a higher risk of being diagnosed with osteoporosis [IRR 1.45(95%CI 1.38–1.53)], osteopenia [IRR 1.17(95%CI 1.09–1.26)], and fragility fracture [IRR 1.50(95%CI 1.44–1.56)] compared to those living in the least deprived areas, but smaller differences were seen in women.

**Conclusion:**

Use of fracture risk assessment tools may enhance the detection of osteoporosis cases in primary care. Further research is needed on the effect of social deprivation on diagnosis of osteoporosis and fractures.

**Supplementary Information:**

The online version contains supplementary material available at 10.1007/s00198-023-06739-1.

## Introduction

Osteoporosis leads to nearly 9 million fractures annually worldwide [1], and over 300,000 fragility fractures in the UK every year [2]. Fragility fractures result from mechanical forces that would not ordinarily result in fracture, known as low-level trauma. The most common sites of osteoporotic fractures are the hip, vertebrae, forearm, but also the pelvis, humerus, and ribs [3]. The impact of hip fractures alone is high, with a 30-day mortality rate of 6.5% [4] and costing an estimated £3.5 billion in the UK in 2010, projected to rise to £5.5 billion per year by 2025 [5]. The total direct cost of osteoporotic fractures in the EU was €56.9 billion in 2019, with hip fractures estimated to account for 57%, vertebral fractures for 10%, distal forearm fractures for 2% and others for 32% of the total costs [6].

Despite the availability of effective treatments [7–10], a great challenge remains the early diagnosis of osteoporosis and timely detection and management of increased risk of fragility fracture before a fracture occurs. According to UK NICE guidelines [11], General Practitioners (GPs) are expected to identify people at high risk of fragility fractures. Although recommended screening tools (e.g. QFracture [12] and FRAX [13]) to estimate the 10-year risk of osteoporotic fracture, as well as Bone Mineral Density (BMD) scans using DXA (dual-energy X-ray absorptiometry), are used in clinical practice, there is no nationwide systematic screening programme for osteoporosis in primary care in the UK, and implementation of fracture risk assessment may vary across practices and geographical regions. GPs’ increasingly busy workload in combination with a lack of public awareness [14] can lead to underdiagnosis and undertreatment of osteoporosis, and subsequent fragility fractures, some of which could be prevented.

The incidence of fragility fractures and in particular hip fractures, which are associated with the highest mortality and disability rates [6], has been a subject of epidemiological research, with considerably heterogeneous results reported in different countries globally [10, 15]. Research on the epidemiology of recorded osteoporosis diagnosis however is scarce and there is no recently published population-based data on the incidence of osteoporosis diagnosis not defined by fracture. Furthermore, there is a lack of data on the incidence of osteopenia from cohort studies, which may reflect a lack of focus on prevention. Although female sex and older age are well-known risk factors for osteoporosis, the role of other demographic characteristics is less known. A recent systematic review demonstrated an association between social deprivation and fragility fractures [16], but the link between social deprivation and a diagnosis of osteoporosis or osteopenia has not been investigated. Hence, we carried out this research study using routinely collected primary care data, aiming to understand how osteoporosis, osteopenia and fragility fractures are recorded in UK primary care and explore trends in observed incidence rates by sociodemographic characteristics. Understanding the patterns of recorded diagnosis of osteoporosis is essential for the design and delivery of public health and community interventions for the prevention of fragility fractures in older people.

The objectives of the present study were: a) to estimate the incidence of a recorded diagnosis of osteoporosis, osteopenia, and fragility fracture in people aged ≥ 50 in the UK; b) to explore time trends in the recording of osteoporosis, osteopenia, and fragility fracture in the UK; c) to describe any differences in incidence rates according to age, sex, and social deprivation.

## Methods

### Data source

We used de-identified data provided by patients as a part of their routine primary care (IQVIA Medical Research Database (IMRD), incorporating data from THIN (The Health Improvement Network), a Cegedim database. Approximately 98% of the UK population is registered with a GP [17]. THIN is a primary care database of over 20 million patients in the UK [18]. GPs record medical diagnoses and symptoms using the Read classification system [19]. Diagnostic Read codes are entered by GPs for conditions that can be diagnosed either in primary care or in secondary care. For example, osteoporosis and osteopenia are often diagnosed in primary care based on DXA bone density scan, and the diagnosis is entered to the patient’s record following review of the result by the GP. They can also (less often) be diagnosed in secondary care (for example if a patient is on medication that increase their risk of osteoporosis and are being monitored by a specialist). In the latter case a diagnostic Read code will be entered to the patient’s primary care record by reviewing the information provided in the clinic letter sent to the GP. Fragility fractures are usually diagnosed in Emergency Departments (as they are often acute, and an x-ray is required), and this information is sent electronically to the patient’s GP and it is subsequently entered as a Read code upon review of the discharge letter. Similarly, if a patient is hospitalised for a major osteoporotic fracture (e.g. hip fracture) the discharge summary containing the Read code is shared with the patient’s GP, and the code is subsequently entered on to the primary care record.

All data in THIN are fully anonymized and they are considered to be representative of the UK population in terms of age, sex, practice size and geographical distribution [20]. A measure of social deprivation recorded as quintiles of Townsend scores is also provided. The Townsend deprivation score is an area-based measure, incorporating unemployment, non-car ownership, non-home ownership and household overcrowding combined, based on an individual’s post (zip) code [21]. We excluded practices that had no linked Townsend data to reduce missing data.

### Study design

Longitudinal population-based cohort study.

### Study population and period

All patients aged 50–99 years registered with participating practices in the THIN database between 1/1/2000 and 31/12/2018 were included. We excluded practices that did not meet criteria for standard quality indicators used in the database, i.e. Acceptable Mortality Reporting (AMR) [22] and Acceptable Computer Usage (ACU) recording [23] during the study period. Study entry was defined as the latest date of patient’s registration with the practice, when they turned 50 years old, or 1/1/2000. The end of the study period was the earliest of the patient’s date of death, the patient’s transfer out of the practice or the last date the practice contributed data to THIN.

### Definition of variables

The outcome variables were: osteoporosis; osteopenia; fragility fracture (defined by a diagnostic Read code entered on the patient’s record) (Appendix Tables 4, 5, and 6). Additional subgroup analyses of first occurrence of a fragility fracture were conducted by site: a) hip; b) vertebrae; c) other, including wrist/radius, pelvic, humerus, ribs, or unspecified site generically coded as fragility fracture. The covariates age, sex and Townsend quintile of social deprivation were included in the analyses as categorical variables.

### Incidence

The date of the first recording of the event in the medical records was used as the date of diagnosis, and therefore incidence rates were estimated based on the number of first recorded episodes. We performed a Lewis plot [24], based on which we excluded events that were diagnosed in the first 6 months from registration with the practice, as they were more likely to represent prevalent cases.

### Statistical analysis

The incidence rate (IR) of osteoporosis, osteopenia, and fragility fractures was estimated per 10,000 person-years (PY) at risk. This was calculated by adding the number of patients with a first recording of diagnostic Read code for osteoporosis, osteopenia, or fragility fracture during the period 2000–2018, and then dividing this number by the total person-years of follow-up for all patient records for this period. We determined incidence rates per age group, sex, social deprivation, and calendar year of diagnosis. Poisson regression was used to compare incidence rates and provide adjusted Incidence Rate Ratios (IRR) by age, sex, social deprivation, and calendar year. Likelihood ratio (LR) tests were performed to explore interactions between covariates. A fixed effects Poisson model was compared against a mixed effects Poisson model using GP practice as a random intercept to assess potential clustering by practice. Statistical analyses were conducted using statistical software Stata 17 (StataCorp).

### Ethical approval

The study protocol was approved by IQVIA Scientific Review Committee (SRC) (Ref. 21SRC011).

## Results

A cohort of more than 3 million people aged 50–99 years from a total of 688 GP practices participating in THIN were included in the analysis. Across all three outcomes, there was a significant interaction between age and sex (p < 0.001) (Suppl. Graph S1, S2, S3), therefore results are presented separately in men and women. Clustering by practice had a significant effect and was therefore included in the adjusted model (Tables 1, 2, 3).

**Table 1:** Crude and adjusted* incidence rates of Osteoporosis diagnosis stratified by sex (2000–2018) (N = 3,275,716) (Men N = 1,587,653; Women N = 1,688,063)

Age (years)	Men – Crude IR per 10,000 PY (95%CI)	Women – Crude IR per 10,000 PY (95%CI)	Men – Adjusted* IRR (95%CI)	Women – Adjusted* IRR (95%CI)
All ages	15.28 (15.06–15.51)	79.82 (79.32–80.31)	1 (Ref.)	4.92 (4.84–5.00)
50–54	4.50 (4.24–4.77)	22.99 (22.38–23.61)	1 (Ref.)	1 (Ref.)
55–59	6.64 (6.30–6.98)	42.44 (41.58–43.32)	1.49 (1.38–1.61)	1.85 (1.79–1.91)
60–64	10.10 (9.66–10.55)	63.50 (62.38–64.63)	2.26 (2.10–2.43)	2.77 (2.68–2.86)
65–69	14.68 (14.11–15.27)	87.27 (85.86–88.70)	3.26 (3.03–3.50)	3.81 (3.69–3.93)
70–74	21.19 (20.42–21.98)	113.32(111.56–115.11)	4.75 (4.43–5.09)	4.96 (4.81–5.12)
75–79	30.84 (29.77–31.94)	143.20 (141.01–145.41)	6.93 (6.47–7.42)	6.28 (6.09–6.48)
80–84	40.84 (39.33–42.40)	153.20 (150.61–155.83)	9.14 (8.51–9.80)	6.73 (6.52–6.95)
85–89	49.51 (47.15–51.97)	153.39 (150.11–156.72)	11.02 (10.21–11.90)	6.75 (6.52–6.99)
90–99	50.47 (46.66–54.52)	118.62 (114.81–122.53)	11.10 (10.07–12.24)	5.22 (5.00–5.44)
Townsend quintile	Men – Crude IR per 10,000 PY (95%CI)	Women—Crude IR per 10,000 PY (95%CI)	Men – Adjusted* IRR (95%CI)	Women – Adjusted* IRR (95%CI)
1 (least deprived)	12.92 (12.54–13.31)	73.56 (72.65–74.48)	1 (Ref.)	1 (Ref.)
2	14.10 (13.67–14.54)	76.98 (75.99–77.98)	1.03 (0.98–1.07)	0.97 (0.96–0.99)
3	15.54 (15.05–16.05)	80.85 (79.76–81.95)	1.12 (1.07–1.17)	0.99 (0.97–1.01)
4	16.86 (16.29–17.45)	85.17 (83.92–86.43)	1.21 (1.15–1.27)	1.01 (0.99–1.03)
5 (most deprived)	21.38 (20.58–22.21)	91.90 (90.27–93.55)	1.45 (1.38–1.53)	1.04 (1.01–1.06)
Year	Men – Crude IR per 10,000 PY (95%CI)	Women – Crude IR per 10,000 PY (95%CI)	Men – Adjusted* IRR (95%CI)	Women – Adjusted* IRR (95%CI)
2000	9.62 (8.64–10.68)	66.01 (63.53–68.56)	1 (Ref.)	1 (Ref.)
2001	10.30 (9.37–11.29)	70.07 (67.73–72.47)	1.08 (0.94–1.24)	1.08 (1.03–1.14)
2002	11.18 (10.29–12.13)	74.27 (72.06–76.54)	1.17 (1.03–1.34)	1.15 (1.09–1.20)
2003	12.87 (11.96–13.82)	87.08 (84.80–89.41)	1.33 (1.17–1.51)	1.34 (1.28–1.41)
2004	12.76 (11.90–13.67)	83.02 (80.88–85.20)	1.31 (1.16–1.49)	1.28 (1.22–1.34)
2005	14.04 (13.16–14.96)	84.90 (82.79–87.04)	1.43 (1.26–1.62)	1.31 (1.25–1.37)
2006	14.26 (13.39–15.17)	82.74 (80.69–84.83)	1.45 (1.29–1.64)	1.29 (1.23–1.35)
2007	15.02 (14.14–15.93)	84.47 (82.42–86.55)	1.53 (1.35–1.72)	1.32 (1.26–1.38)
2008	14.29 (13.45–15.18)	80.70 (78.72–82.72)	1.45 (1.28–1.64)	1.26 (1.20–1.31)
2009	15.90 (15.01–16.82)	92.17 (90.06–94.31)	1.60 (1.42–1.81)	1.44 (1.38–1.50)
2010	13.76 (12.93–14.62)	79.20 (77.22–81.21)	1.38 (1.22–1.56)	1.24 (1.18–1.30)
2011	13.75 (12.93–14.61)	71.37 (69.51–73.26)	1.38 (1.22–1.55)	1.12 (1.07–1.17)
2012	17.35 (16.43–18.31)	76.46 (74.55–78.42)	1.73 (1.54–1.95)	1.20 (1.14–1.25)
2013	19.69 (18.70–20.73)	81.22 (79.21–83.27)	1.94 (1.72–2.18)	1.26 (1.21–1.32)
2014	20.00 (18.98–21.07)	84.83 (82.73–86.98)	1.93 (1.72–2.18)	1.31 (1.25–1.37)
2015	19.94 (18.84–21.09)	82.10 (79.86–84.38)	1.89 (1.67–2.13)	1.25 (1.19–1.31)
2016	19.06 (17.88–20.29)	77.97 (75.58–80.42)	1.77 (1.56–2.00)	1.16 (1.11–1.22)
2017	16.57 (15.38–17.82)	71.34 (68.87–73.88)	1.54 (1.36–1.76)	1.06 (1.01–1.12)
2018	15.62 (14.41–16.91)	67.41 (64.87–70.03)	1.43 (1.25–1.63)	0.98 (0.93–1.04)

*Adjusted for age, Townsend quintile of social deprivation, calendar year, and clustering by practice effect

**Table 2:** Crude and adjusted* incidence rates of Osteopenia diagnosis stratified by sex (2000–2018) (N = 3,326,188) (Men N = 1,593,152; Women N = 1,733,036)

Age (years)	Men – Crude IR per 10,000 PY (95%CI)	Women – Crude IR per 10,000 PY (95%CI)	Men – Adjusted* IRR (95%CI)	Women – Adjusted* IRR (95%CI)
All ages	8.65 (8.48–8.82)	45.10 (44.74–45.47)	1 (Ref.)	5.33 (5.22–5.45)
50–54	3.82 (3.58–4.07)	26.70 (26.05–27.37)	1 (Ref.)	1 (Ref.)
55–59	5.56 (5.25–5.87)	38.55 (37.73–39.38)	1.49 (1.37–1.62)	1.47 (1.42–1.52)
60–64	7.70 (7.32–8.09)	51.76 (50.76–52.78)	2.03 (1.87–2.21)	1.96 (1.90–2.02)
65–69	10.22 (9.75–10.71)	61.15 (59.99–62.32)	2.68 (2.47–2.90)	2.33 (2.25–2.40)
70–74	12.92 (12.32–13.53)	63.48 (62.20–64.78)	3.44 (3.17–3.72)	2.47 (2.39–2.55)
75–79	14.72 (13.99–15.48)	56.57 (55.26–57.91)	3.96 (3.65–4.30)	2.24 (2.16–2.31)
80–84	14.33 (13.44–15.26)	41.18 (39.92–42.47)	3.83 (3.50–4.19)	1.63 (1.57–1.70)
85–89	13.71 (12.49–15.02)	28.40 (27.09–29.75)	3.61 (3.23–4.04)	1.11 (1.05–1.17)
90–99	10.42 (8.75–12.32)	16.39 (15.09–17.78)	2.74 (2.29–3.27)	0.63 (0.58–0.69)
Townsend quintile	Men – Crude IR per 10,000 PY (95%CI)	Women – Crude IR per 10,000 PY (95%CI)	Men – Adjusted* IRR (95%CI)	Women – Adjusted* IRR (95%CI)
1 (least deprived)	7.60 (7.31–7.90)	46.38 (45.67–47.10)	1 (Ref.)	1 (Ref.)
2	8.12 (7.80–8.46)	44.90 (44.16–45.65)	1.01 (0.96–1.07)	0.97 (0.94–0.99)
3	8.87 (8.50–9.25)	45.19 (44.39–46.00)	1.06 (1.00–1.12)	0.93 (0.90–0.95)
4	9.46 (9.03–9.90)	43.40 (42.53–44.29)	1.10 (1.03–1.18)	0.89 (0.87–0.91)
5 (most deprived)	10.96 (10.39–11.56)	44.79 (43.68–45.92)	1.17 (1.09–1.26)	0.87 (0.84–0.90)
Year	Men – Crude IR per 10,000 PY (95%CI)	Women – Crude IR per 10,000 PY (95%CI)	Men – Adjusted* IRR (95%CI)	Women – Adjusted* IRR (95%CI)
2000	1.36 (1.01–1.80)	9.17 (8.28–10.13)	1 (Ref.)	1 (Ref.)
2001	2.47 (2.03–2.98)	17.71 (16.57–18.91)	1.82 (1.30–2.54)	1.94 (1.72–2.19)
2002	3.34 (2.86–3.87)	23.23 (22.02–24.48)	2.43 (1.77–3.33)	2.54 (2.27–2.85)
2003	3.28 (2.83–3.77)	32.47 (31.11–33.87)	2.40 (1.75–3.27)	3.60 (3.23–4.01)
2004	4.57 (4.06–5.12)	33.29 (31.97–34.65)	3.36 (2.49–4.53)	3.71 (3.33–4.13)
2005	6.17 (5.59–6.79)	36.50 (35.15–37.88)	4.53 (3.38–6.08)	4.08 (3.67–4.54)
2006	6.62 (6.03–7.25)	38.23 (36.87–39.63)	4.84 (3.62–6.49)	4.28 (3.84–4.75)
2007	7.30 (6.70–7.95)	41.30 (39.90–42.73)	5.34 (3.99–7.14)	4.62 (4.15–5.13)
2008	8.58 (7.93–9.27)	45.85 (44.40–47.35)	6.23 (4.67–8.32)	5.09 (4.58–5.65)
2009	8.18 (7.55–8.85)	58.82 (57.18–60.50)	5.90 (4.42–7.88)	6.52 (5.87–7.23)
2010	9.95 (9.25–10.69)	51.29 (49.74–52.88)	7.15 (5.36–9.52)	5.66 (5.10–6.28)
2011	10.13 (9.43–10.88)	48.56 (47.06–50.10)	7.20 (5.40–9.59)	5.31 (4.78–5.90)
2012	10.98 (10.25–11.75)	54.70 (53.11–56.32)	7.75 (5.82–10.31)	5.98 (5.38–6.63)
2013	11.68 (10.92–12.48)	62.41 (60.69–64.17)	8.19 (6.15–10.90)	6.78 (6.11–7.52)
2014	12.31 (11.51–13.15)	58.91 (57.19–60.67)	8.53 (6.41–11.35)	6.35 (5.72–7.05)
2015	12.39 (11.53–13.30)	55.69 (53.88–57.54)	8.38 (6.29–11.17)	5.95 (5.36–6.62)
2016	14.30 (13.28–15.37)	58.75 (56.71–60.85)	9.42 (7.07–12.57)	6.18 (5.56–6.87)
2017	13.75 (12.68–14.90)	54.07 (51.95–56.26)	8.83 (6.61–11.80)	5.58 (5.01–6.22)
2018	14.25 (13.10–15.48)	54.85 (52.58–57.18)	8.92 (6.67–11.94)	5.62 (5.04–6.27)

*Adjusted for age, Townsend quintile of social deprivation, calendar year, and clustering by practice effect

**Table 3:** Crude and adjusted* incidence rates of fragility fracture stratified by sex (2000–2018) (Men N = 1,537,217; Women N = 1,654,625) (N = 3,191,842)

Age (years)	Men – IR per 10,000 PY (95%CI)	Women – IR per 10,000 PY (95%CI)	Men – Adjusted IRR (95%CI)	Women – Adjusted IRR (95%CI)
All ages	28.72 (28.41–29.03)	82.01 (81.50–82.51)	1 (Ref.)	2.55 (2.52–2.58)
50–54	14.25 (13.77–14.74)	23.83 (23.21–24.47)	1 (Ref.)	1 (Ref.)
55–59	15.41 (14.89–15.94)	36.61 (35.81–37.43)	1.09 (1.04–1.15)	1.55 (1.50–1.61)
60–64	18.17 (17.57–18.78)	49.80 (48.81–50.80)	1.29 (1.23–1.35)	2.09 (2.03–2.16)
65–69	22.42 (21.71–23.16)	66.92 (65.69–68.16)	1.58 (1.50–1.65)	2.81 (2.72–2.90)
70–74	29.95 (29.02–30.90)	90.10 (88.54–91.69)	2.13 (2.03–2.23)	3.84 (3.72–3.96)
75–79	46.04 (44.71–47.40)	132.24 (130.14–134.36)	3.28 (3.14–3.43)	5.68 (5.50–5.85)
80–84	72.27 (70.22–74.37)	191.25 (188.34–194.19)	5.14 (4.92–5.38)	8.26 (8.01–8.51)
85–89	118.37 (114.62–122.21)	259.64 (255.26–264.08)	8.37 (7.99–8.77)	11.18 (10.84–11.54)
90–99	173.89 (166.54–181.48)	322.99 (316.29–329.80)	12.24 (11.59–12.93)	14.03 (13.56–14.51)
Townsend quintile	Men – IR per 10,000 PY (95%CI)	Women – IR per 10,000 PY (95%CI)	Men – Adjusted IRR (95%CI)	Women – Adjusted IRR (95%CI)
1 (least deprived)	24.02 (23.49–24.56)	72.15 (71.25–73.06)	1 (Ref.)	1 (Ref.)
2	27.16 (26.55–27.78)	78.97 (77.96–79.98)	1.08 (1.04–1.11)	1.01 (0.99–1.03)
3	29.21 (28.52–29.91)	83.59 (82.47–84.71)	1.18 (1.14–1.22)	1.05 (1.03–1.07)
4	32.78 (31.96–33.62)	91.61 (90.31–92.93)	1.31 (1.27–1.36)	1.10 (1.08–1.12)
5 (most deprived)	37.82 (36.73–38.94)	96.36 (94.68–98.07)	1.50 (1.44–1.56)	1.12 (1.09–1.14)
Year	Men – IR per 10,000 PY (95%CI)	Women – IR per 10,000 PY (95%CI)	Men – Adjusted IRR (95%CI)	Women – Adjusted IRR (95%CI)
2000	21.47 (19.97–23.05)	60.19 (57.80–62.65)	1 (Ref.)	1 (Ref.)
2001	23.37 (21.94–24.86)	62.52 (60.29–64.81)	1.09 (0.99–1.19)	1.04 (0.98–1.09)
2002	22.32 (21.04–23.66)	66.12 (64.01–68.28)	1.04 (0.95–1.14)	1.09 (1.04–1.15)
2003	24.50 (23.23–25.82)	67.08 (65.06–69.14)	1.15 (1.05–1.25)	1.11 (1.06–1.17)
2004	24.44 (23.22–25.69)	66.91 (64.98–68.88)	1.15 (1.06–1.26)	1.12 (1.06–1.17)
2005	23.69 (22.53–24.89)	69.63 (67.71–71.58)	1.11 (1.02–1.21)	1.16 (1.11–1.22)
2006	23.25 (22.12–24.42)	66.97 (65.12–68.86)	1.09 (1.00–1.19)	1.12 (1.07–1.18)
2007	24.90 (23.75–26.10)	68.35 (66.51–70.24)	1.16 (1.06–1.26)	1.15 (1.09–1.20)
2008	24.38 (23.25–25.54)	78.24 (76.28–80.23)	1.13 (1.04–1.23)	1.31 (1.25–1.38)
2009	27.90 (26.70–29.14)	99.20 (97.01–101.43)	1.29 (1.19–1.40)	1.68 (1.60–1.76)
2010	29.89 (28.64–31.18)	92.23 (90.09–94.40)	1.37 (1.26–1.49)	1.56 (1.49–1.64)
2011	29.19 (27.97–30.46)	88.41 (86.33–90.52)	1.33 (1.22–1.44)	1.49 (1.42–1.56)
2012	34.40 (33.08–35.76)	105.07 (102.82–107.36)	1.55 (1.43–1.68)	1.77 (1.69–1.85)
2013	36.56 (35.18–37.99)	100.47 (98.23–102.75)	1.64 (1.51–1.78)	1.70 (1.62–1.78)
2014	36.69 (35.27–38.15)	99.08 (96.79–101.41)	1.63 (1.50–1.77)	1.66 (1.59–1.74)
2015	35.41 (33.90–36.96)	95.38 (92.96–97.85)	1.57 (1.44–1.71)	1.60 (1.52–1.67)
2016	34.38 (32.76–36.07)	85.13 (82.62–87.70)	1.53 (1.40–1.67)	1.43 (1.36–1.50)
2017	33.97 (32.22–35.78)	84.09 (81.38–86.85)	1.49 (1.36–1.63)	1.39 (1.32–1.47)
2018	32.12 (30.33–33.98)	77.80 (75.06–80.63)	1.39 (1.27–1.53)	1.28 (1.21–1.35)

*Adjusted for age, Townsend quintile of social deprivation, calendar year, and clustering by practice effect

### Osteoporosis

**Fig. 1:**
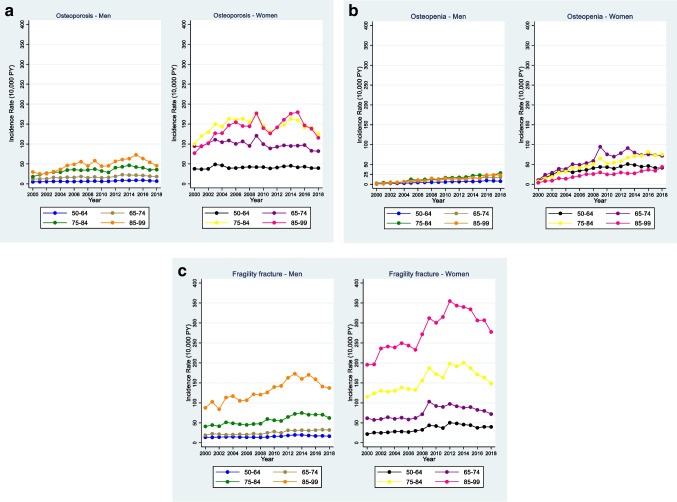
Incidence graphs for recorded diagnosis of osteoporosis, osteopenia, and fragility fractures in men and women aged ≥ 50 years in the UK (2000–2018). Graph 1A: Incidence of osteoporosis diagnosis in men and women (2000–2018). Graph 1B: Incidence of osteopenia diagnosis in men and women (2000–2018). Graph 1C: Fragility fracture incidence in men and women (2000–2018)

The overall crude incidence of osteoporosis diagnosis was significantly higher in women, 79.82 vs. 15.28 in men per 10,000 PY. A peak of recorded osteoporosis diagnosis was observed in 2009 in women, followed by a period of increased incidence between 2013–2015 in both men and women (Fig. 1. Graph 1A). The incidence of osteoporosis diagnosis increased with age: it was lowest in the age group 50–54 in both men and women, and it was highest in men 90-99y and in women 85-89y. The overall adjusted risk of osteoporosis diagnosis across all ages was 4.9 times higher in women vs. men [IRR 4.92 (95%CI 4.84–5.00)]. The crude IR of osteoporosis diagnosis increased from 2009 onwards in women, and from 2012 onwards in men. The year-specific adjusted IRR of recorded osteoporosis was highest in 2009 in women [IRR 1.44 (95%CI 1.38–1.50)], whereas in men it was highest in 2013 and 2014 [IRR 1.94 (95%CI 1.72–2.18)] compared to the reference (year 2000). In the adjusted model, older men living in most deprived areas were almost 1.5 times more likely to be diagnosed with osteoporosis [IRR 1.45 (95% 1.38–1.53)] compared to men in the least deprived areas, whereas the risk of osteoporosis diagnosis was only borderline increased for older women living in most deprived areas [IRR 1.04 (1.01–1.06)] compared to women in the least deprived areas (Table 1).

### Osteopenia

Women were more likely to be diagnosed with osteopenia compared to men, at any age. The overall IR of osteopenia diagnosis was 45.10 in women vs. 8.65 in men per 10,000 PY. The incidence of osteopenia diagnosis was very low at the start of the study period, and it progressively increased, reaching a peak in women in 2009, and a second peak in 2013. In women however osteopenia IRs were lowest in those aged 85-99y (Fig. 1.Graph 1B). The adjusted IRR of osteopenia diagnosis in women was 5.33 (95%CI 5.22–5.45) compared to men. In the adjusted model, men in the most deprived areas had 1.2 times higher risk of being diagnosed with osteopenia [IRR 1.17 (95% 1.03–1.18)] compared to men in the least deprived areas, whereas there was no significant effect of deprivation on osteopenia diagnosis in women (Table 2).

### Fragility fractures

The incidence of recorded fragility fractures increased with age and an increasing trend was observed during the study period. The overall crude IR in men was 28.72 vs. 82.01 in women per 10,000 PY. The age-specific IR of fragility fractures was highest in the age group 90-99y for both sexes. The crude IR of fragility fracture increased from 2009 onwards in women and from 2012 onwards in men. The year-specific adjusted IRR of fragility fracture in women was highest in 2012 [IRR 1.77 (95%CI 1.69–1.85)], whereas in men it was highest in 2013 [IRR 1.64 (95%CI 1.51–1.78)] compared to the reference (year 2000). The adjusted IRR of fragility fracture in women (across all age groups) was 2.55 (95%CI 2.52–2.58) compared to men. In the adjusted model, men in most deprived areas had 1.5 times higher risk of sustaining a fragility fracture [IRR 1.50 (95%CI 1.44–1.56)] compared to men in the least deprived areas, whereas women in most deprived areas were almost 1.1 times more likely to have a fragility fracture compared to women in the least deprived areas [IRR 1.12 (95%CI 1.09–1.14)] (Table 3).

**Fig. 2:**
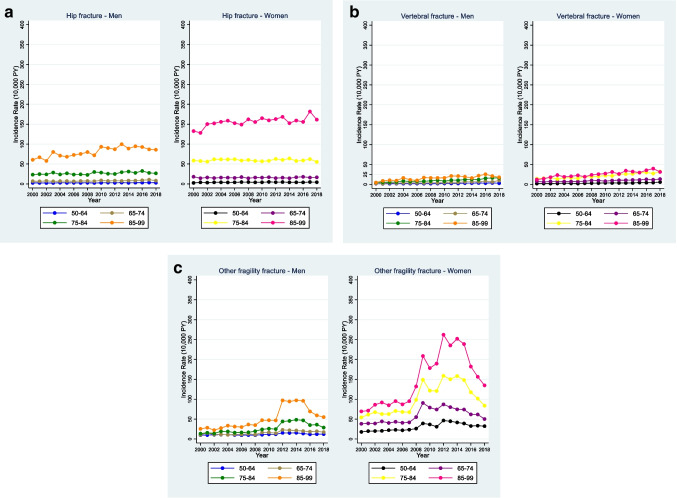
Incidence graphs for recorded diagnosis of hip, vertebral, and other fragility fractures in men and women aged ≥ 50 years in the UK (2000–2018). Graph 2A: Hip fracture incidence in men and women (2000–2018). Graph 2B: Vertebral fracture incidence in men and women (2000–2018). Graph 2C: Other fragility fracture incidence in men and women (2000–2018)

Additional analyses by fracture site demonstrated a crude IR of hip fracture 10.44 in men and 27.30 in women per 10,000 PY. The recorded diagnosis of hip fracture was stable during the study period, and the risk of suffering a hip fracture was extremely high in the oldest old (90-99y), compared to 50-54y [men IRR 60.16 (95%CI 54.15–66.85); women IRR 92.33 (95%CI 84.26–101.17)] (Fig. 2. Graph 2A). In the adjusted model, social deprivation increased the risk of hip fracture in both men [IRR 1.70 (95%CI 1.60–1.81)] and women [IRR 1.20 (95%CI 1.15–1.25)] (Suppl. Table S1).

Rates of first recorded vertebral fracture were very low compared to fractures at other sites, with an overall IR of vertebral fracture 4.60 in men and 9.47 in women per 10,000 PY. There was however a slight increase in incidence time trend for older age groups (Fig. 2. Graph 2B). In the adjusted model, the effect of social deprivation on vertebral fracture was significant in both men [IRR 1.46 (95%CI 1.32–1.61)] and women [IRR 1.26 (95%CI 1.18–1.35)] (Suppl. Table S2).

The IR of a first recorded other fragility fracture was higher compared to hip fracture; men: IR 16.75, women: 58.73, per 10,000 PY. The incidence rate increased from 2009 onwards, for people aged ≥ 75y, and it reached a peak during 2012–2014 (Fig. 2. Graph 2C). In the adjusted model, social deprivation increased the risk of other fragility fracture in both men [IRR 1.46 (95%CI 1.39–1.54)] and women [IRR 1.09 (95%CI 1.06–1.12)] (Suppl. Table S3).

## Discussion

In this study we report the incidence of a diagnosis of osteoporosis, osteopenia and fragility fracture as recorded in primary care. Osteoporosis and fragility fractures were found to be more commonly diagnosed in women and older age groups. The incidence of recorded osteopenia was in general lower than expected, although it was higher in women, and it decreased with advanced age in both sexes. Social deprivation was independently associated with higher risk of osteoporosis and osteopenia diagnosis and fragility fractures in men, whereas smaller differences were seen in women.

We were unable to identify any comparable population-based studies reporting the incidence of osteoporosis diagnosis not defined by a fracture, based on routinely collected primary care data. The available literature reports prevalence and not incidence of osteoporosis. The prevalence of osteoporosis varies across studies, depending on the country, population sample, age, and case finding method [25]; it has been reported 10.3% in people aged ≥ 50 in the US [26], 24% in women in their 7^th^ decade in a UK study [27], and 30–40% in women and 10–20% in men aged > 50 in China [28]. Prevalence of osteopenia (based on DXA) was reported to be higher, 43.9% [26] and 49% [27] from US and UK studies respectively. In our study we found lower than expected rates of osteopenia diagnosis, given that in other studies the prevalence of osteopenia was higher than that of osteoporosis, and we would therefore expect incidence rates of osteopenia to be respectively higher. However, these studies have a different design compared to our study, as they have used screening to define osteoporosis or osteopenia in smaller population samples, whereas we have studied the incidence of recorded osteoporosis and osteopenia based on diagnostic Read codes in a very large dataset of routinely collected data.

Our study found that the incidence of osteoporosis diagnosis and fragility fractures increased from 2009 onwards in women, and from 2012 onwards in men. This could be explained by the introduction of fracture risk assessment tools around that time. More specifically, the FRAX score was introduced in 2008 [13], whereas the validation paper of the original QFracture was published in 2009 [29]. Importantly, the introduction of fracture risk assessment tools was part of the comprehensive guidelines on the prevention and treatment of osteoporosis (The National Osteoporosis Guideline Group), which were first introduced in 2008 [30], followed by later updates in subsequent years. Publicity of the new fracture risk screening tools might have triggered an interest of GPs in using those, leading to identification of more cases. QFracture was subsequently updated in 2012 [12], and osteoporosis was introduced to the Quality and Outcomes Framework for GPs in 2012–13 [31]. As part of this incentives’ scheme, GPs are rewarded with points for people aged ≥ 50 with a diagnosis of osteoporosis confirmed on DXA, who have not sustained a fragility fracture before the age of 75, and people aged ≥ 75 with a record of fragility fracture and a diagnosis of osteoporosis [32]. Nevertheless, despite the peak in the recorded diagnosis, we did not see a corresponding reduction in subsequent fractures in the following years. We therefore need to understand if diagnosis is triggering appropriate treatment. A review of quality measures and quality improvement initiatives for osteoporosis in the US found a gradual improvement in osteoporosis screening, identification and treatment following fragility fracture (2006–2016), although according to data from population-based studies, performance for these quality measures was lower when reporting was not mandatory [33]. Systematic reviews have shown that the Fracture Liaison Service (FLS) model of care is associated with improvements in rates of bone mineral density testing, initiation of osteoporosis treatment and adherence with treatment in people with fragility fractures [34].

The lower incidence of recorded osteopenia and osteoporosis diagnosis in older men contrasts with the high incidence of fragility fractures in this group. This finding implies that there is a gap in prevention, which is more prominent in men. Traditionally osteoporosis screening has been targeted at women, and bone health is a domain commonly overlooked in men. Similarly, the oldest old seem to have very low rates of osteopenia diagnosis, but very high rates of fragility fractures. This is likely to represent a very low number of referrals for DXA scans in the oldest old, possibly due to a reluctance of patients, relatives, or healthcare professionals, on the grounds of other health priorities, multimorbidity and frailty. There is also debate about the value of treatment in people with a low life expectancy and quality of life, e.g. people with dementia, polypharmacy, and greater risk of adverse effects.

In our study we found a slightly lower incidence rate of fragility fracture and hip fracture compared to that reported in a UK study using Clinical Practice Research Datalink (CPRD) data (1988–2012) [35]. Interestingly, the incidence rate of vertebral fracture in people above 50 in that study [35] was the same as in our study. It is worth noting that the actual incidence of vertebral fractures is likely to be higher, as they are often asymptomatic, they can therefore be missed from diagnosis, although recent techniques can improve detection [36, 37]. The majority of other studies reporting the incidence of hip and vertebral fractures have been conducted in women, using convenience, and not population-based samples [38], and with case finding in secondary care [38, 39] or via surveys [40]. The incidence of hip fractures has been found to be lower in Eastern countries [38, 39, 41–43], whereas it has been reported to be higher in Northern Europe [6, 37, 44].

Temporal trends in the incidence of hip fracture in Portugal were found to be affected by socioeconomic inequalities which were more marked in women aged 65–79 [45]. A systematic review of observational studies showed that low socioeconomic status measured at the individual level (education, income, occupation, co-habiting) was associated with an increased risk of both hip and non-hip fragility fracture [RR 1.27 (95%CI 1.12–1.44)], whereas the use of area-based measures of deprivation did not provide a statistically significant association [16]. In our study social deprivation measured using the area-based Townsend index was significantly and independently associated with osteoporosis diagnosis and fragility fractures in both sexes and with osteopenia diagnosis in men.

The present study has strengths and limitations. The main strength is the rigorous methodology, using nationally representative, real-world data. This is, to our knowledge, the first population-based study estimating incidence of recorded diagnosis of osteoporosis in primary care. A limitation is that analyses were based on Read codes as they were recorded by GPs, which can be influenced by various factors, e.g. GP workload, length of consultation, allocation of additional time for administrative tasks, and different coding behaviours amongst GPs or across different practices. It is therefore possible that some cases of osteoporosis are identified and treated without a diagnosis being formally coded. We did not have access to DXA results to explore this, and the incidence of osteoporosis, and especially osteopenia, is likely to be underestimated. Despite the fact that the actual osteopenia incidence rates in this population of people above 50 are probably much higher, the under-recording and under-diagnosis of osteopenia in primary care records is an important finding. Although fragility fractures are generally more likely to be identified (with the exception of vertebral fractures) and therefore coded in patients’ electronic records, it is still possible that a proportion of these fractures may not be coded in the primary care records, for example if data is not transcribed fully from secondary care correspondence onto the primary care record or if a fragility fracture occurs in the community but a diagnostic Read code is not inserted at the time. Moreover, while analysis of prescriptions of anti-osteoporotic medication was beyond the scope of this project, it is possible that, had prescription data been included in this analysis, we might have identified some additional cases of osteoporosis or fragility fractures which were not captured by diagnostic coding.

There are significant implications arising from this study. Despite the increased incidence of recorded osteoporosis after the introduction of fracture risk screening tools, the incidence of fragility fractures increased over time in men and women, even after accounting for age. Osteopenia appears to be under-diagnosed, which highlights an important gap in early detection and missed opportunity for intervention. We need further research on management and the prescription of treatments to understand why age-adjusted fragility fracture rates are rising and examine if increased recording of osteoporosis translates into better management. The association of the area-based Townsend index of deprivation with osteoporosis and fragility fractures, which is more pronounced in men, warrants further research to understand the reasons for this. More specifically we need to explore any socioeconomic inequalities in screening for osteoporosis and subsequent management, as well as prescription of bone-sparing medication for older people with fragility fractures.

## Conclusion

The identification and recording of a diagnosis of osteoporosis, osteopenia, or fragility fracture may have improved due to the introduction of osteoporotic fracture risk assessment tools as well as incentivization schemes in primary care. As expected, female sex and advanced age were associated with higher incidence of osteoporosis and fragility fractures in this study. We also found significantly increased risk of osteoporosis, osteopenia and fragility fractures in men living in deprived areas. This work has identified that further research is needed on the effect of social deprivation on diagnosis of osteoporosis and fractures.

### Electronic supplementary material

Below is the link to the electronic supplementary material.

Supplementary file1 (DOCX 54.9 KB)

## Appendix

Table

**Table 4:** Read code list for Osteoporosis

Read code	Description
66a9.00	Osteoporosis—falls prevention
66aA.00	Osteoporosis—treatment response
66aB.00	Osteoporosis—no treatment response
9hP..00	Exception reporting: osteoporosis quality indicators
N330.00	Osteoporosis
N330000	Osteoporosis, unspecified
N330100	Senile osteoporosis
N330200	Postmenopausal osteoporosis
N330300	Idiopathic osteoporosis
N330400	Dissuse osteoporosis
N337000	Disuse atrophy of bone
N330600	Postoophorectomy osteoporosis
N330700	Postsurgical malabsorption osteoporosis
N330800	Localized osteoporosis—Lequesne
N330900	Osteoporosis in multiple myelomatosis
N330A00	Osteoporosis in endocrine disorders
N330C00	Osteoporosis localized to spine
N330D00	Osteoporosis due to corticosteroids
N331D00	Collapsed vertebra NOS
N331200	Postoophorectomy osteoporosis with pathological fracture
N331300	Osteoporosis of disuse with pathological fracture
N331400	Postsurgical malabsorption osteoporosis with pathological fracture
N331500	Drug-induced osteoporosis with pathological fracture
N331600	Idiopathic osteoporosis with pathological fracture
N331800	Osteoporosis + pathological fracture lumbar vertebrae
N331900	Osteoporosis + pathological fracture thoracic vertebrae
N331A00	Osteoporosis + pathological fracture cervical vertebrae
N331B00	Postmenopausal osteoporosis with pathological fracture
N331H00	Collapse of cervical vertebra due to osteoporosis
N331J00	Collapse of lumbar vertebra due to osteoporosis
N331K00	Collapse of thoracic vertebra due to osteoporosis
N331L00	Collapse of vertebra due to osteoporosis NOS
NyuB000	[X]Other osteoporosis with pathological fracture
NyuB100	[X]Other osteoporosis
NyuB200	[X]Osteoporosis in other disorders classified elsewhere
NyuB800	[X]Unspecified osteoporosis with pathological fracture
66a..00	Osteoporosis monitoring
66a2.00	Osteoporosis treatment started
66a5.00	Osteoporosis—no treatment
66a6.00	Osteoporosis—dietary advice
66a7.00	Osteoporosis—dietary assessment
66a8.00	Osteoporosis—exercise advice
9Od0.00	Attends osteoporosis monitoring
9Od2.00	Osteoporosis monitoring default
66aE.00	Refer to osteoporosis specialist
9N0h.00	Seen in osteoporosis clinic
8HTS.00	Referral to osteoporosis clinic
9kj0.00	Bone sparing drug treatment offered for osteoporosis—enhanced services administration
9hP0.00	Excepted from osteoporosis quality indicators: patient unsuitable
9hP1.00	Excepted from osteoporosis quality indicators: informed dissent
N330z00	Osteoporosis NOS
N330500	Drug-induced osteoporosis
N374600	Osteoporotic kyphosis
58EG.00	Hip DXA scan result osteoporotic
58EM.00	Lumbar DXA scan result osteoporotic
58EV.00	Femoral neck DEXA scan result osteoporotic
N330B.00	Vertebral osteoporosis

4,

**Table 5:** Read code list for Osteopenia

Read code	Description
NyuBC00	[X]Osteopenia

5,

**Table 6:** Read code list for Fragility fractures

Read code	Description
7J42600	Primary bedrest stabilisation of spinal fracture
7J42700	Primary collar stabilisation of spinal fracture
7J42900	Primary cast stabilisation of spinal fracture
7J42B00	Primary other external stabilisation of spinal fracture
7J42C00	Revision to bedrest stabilisation of spinal fracture
7J42D00	Revision to collar stabilisation of spinal fracture
7J42G00	Revision to external fixation stabilisation of spinal fracture
7J42J00	Primary closed reduction spinal fracture alone
7J42L00	Primary closed reduction spinal fracture and bedrest stabilisation
7J42M00	Primary closed reduction spinal fracture and skull traction stabilisation
7J42y00	Other specified other reduction of fracture of spine
7J42z00	Other reduction of fracture of spine NOS
7J43.00	Fixation of fracture of spine
7J43000	Primary open reduction spinal fracture and internal fixation with plate
7J43100	Fixation of fracture of spine using Harrington rod
7J43200	Fixation of fracture of spine and skull traction however further qualified
7J43300	Primary open reduction spinal fracture and internal fixation with wire
7J43400	Primary open reduction spinal fracture and internal fixation with rod system
7J43700	Primary open reduction spinal fracture and other internal fixation
7J43900	Revision to open reduction spinal fracture and internal fixation with plate
7J43A00	Revision to open reduction spinal fracture and internal fixation with rod system
7J43C00	Revision to open reduction spinal fracture and internal fixation with internal fixator
7J43E00	Removal of fracture fixation device from spine
7J43y00	Other specified fixation of fracture of spine
7J43z00	Fixation of fracture of spine NOS
7K1D000	Primary open reduction and internal fixation of proximal femoral fracture with screw/nail and plate device
7K1D600	Primary open reduction and internal fixation of proximal femoral fracture with screw/nail device alone
7K1H600	Revision to open reduction and internal fixation of proximal femoral fracture with screw/nail device alone
7K1H800	Revision to open reduction and internal fixation of proximal femoral fracture with screw/nail and plate device
7K1J000	Closed reduction and internal fixation of proximal femoral fracture with screw/nail device alone
7K1J600	Primary internal fixation(without reduction) of proximal femoral fracture with screw/nail and intramedullary device
7K1J800	Revision to internal fixation(without reduction) of proximal femoral fracture with screw/nail device alone
7K1JB00	Primary closed reduction and internal fixation of proximal femoral fracture with screw/nail device alone
7K1JD00	Primary closed reduction and internal fixation of proximal femoral fracture with screw/nail and plate device
7K1L400	Closed reduction of fracture of hip
7K1LL00	Closed reduction of fracture of radius and or ulna
7K1LM00	Closed reduction of fracture of wrist
7P20100	Delivery of rehabilitation for hip fracture
N1y1.00	Fatigue fracture of vertebra
N331D00	Collapsed vertebra NOS
N331F00	Collapse of thoracic vertebra
N331G00	Collapse of lumbar vertebra
N331200	Postoophorectomy osteoporosis with pathological fracture
N331300	Osteoporosis of disuse with pathological fracture
N331400	Postsurgical malabsorption osteoporosis with pathological fracture
N331500	Drug-induced osteoporosis with pathological fracture
N331600	Idiopathic osteoporosis with pathological fracture
N331800	Osteoporosis + pathological fracture lumbar vertebrae
N331900	Osteoporosis + pathological fracture thoracic vertebrae
N331A00	Osteoporosis + pathological fracture cervical vertebrae
N331B00	Postmenopausal osteoporosis with pathological fracture
N331H00	Collapse of cervical vertebra due to osteoporosis
N331J00	Collapse of lumbar vertebra due to osteoporosis
N331K00	Collapse of thoracic vertebra due to osteoporosis
N331L00	Collapse of vertebra due to osteoporosis NOS
N331M00	Fragility fracture due to unspecified osteoporosis
N331N00	Fragility fracture
NyuB000	[X]Other osteoporosis with pathological fracture
S10..00	Fracture of spine without mention of spinal cord injury
S102.00	Closed fracture thoracic vertebra
S102000	Closed fracture thoracic vertebra, burst
S102100	Closed fracture thoracic vertebra, wedge
S102200	Closed fracture thoracic vertebra, spondylolysis
S102300	Closed fracture thoracic vertebra, spinous process
S102400	Closed fracture thoracic vertebra, transverse process
S102500	Closed fracture thoracic vertebra, posterior arch
S102y00	Other specified closed fracture thoracic vertebra
S102z00	Closed fracture thoracic vertebra not otherwise specified
S104.00	Closed fracture lumbar vertebra
S104000	Closed fracture lumbar vertebra, burst
S104100	Closed fracture lumbar vertebra, wedge
S104200	Closed fracture lumbar vertebra, spondylolysis
S104300	Closed fracture lumbar vertebra, spinous process
S104400	Closed fracture lumbar vertebra, transverse process
S104500	Closed fracture lumbar vertebra, posterior arch
S104600	Closed fracture lumbar vertebra, tricolumnar
S106.00	Closed fracture sacrum
S106000	Closed compression fracture sacrum
S106100	Closed vertical fracture of sacrum
S108.00	Closed fracture pelvis, coccyx
S10B.00	Fracture of lumbar spine and pelvis
S10B000	Fracture of lumbar vertebra
S10B100	Fracture of sacrum
S10B200	Fracture of coccyx
S10x.00	Closed fracture of spine, unspecified,
S10z.00	Fracture of spine without mention of spinal cord lesion NOS
S120.00	Closed fracture rib
S120000	Closed fracture of rib, unspecified
S120100	Closed fracture of one rib
S120200	Closed fracture of two ribs
S120300	Closed fracture of three ribs
S127100	Cough fracture of ribs
S120z00	Closed fracture of rib(s) NOS
S127.00	Fracture of rib
S120A00	Cough fracture
S132.00	Closed fracture pubis
S132000	Closed fracture pelvis, single pubic ramus
S132100	Closed fracture pelvis, multiple pubic rami—stable
S132200	Closed fracture pelvis, multiple pubic rami—unstable
S132y00	Other specified closed fracture pubis
S132z00	Closed fracture pubis NOS
S15..00	Fracture of thoracic vertebra
S220.00	Closed fracture of the proximal humerus
S220000	Closed fracture of proximal humerus, unspecified part
S220100	Closed fracture proximal humerus, neck
S220200	Closed fracture of proximal humerus, anatomical neck
S220300	Closed fracture proximal humerus, greater tuberosity
S220400	Closed fracture proximal humerus, head
S220500	Closed fracture of humerus, upper epiphysis
S220600	Closed fracture proximal humerus, three part
S220700	Closed fracture proximal humerus, four part
S220z00	Closed fracture of proximal humerus not otherwise specified
S226.00	Fracture of upper end of humerus
S234.00	Closed fracture of radius and ulna, lower end
S240.00	Closed fracture of carpal bone
S234000	Closed fracture of forearm, lower end, unspecified
S234100	Closed Colles' fracture
S234700	Closed Smith's fracture
S234200	Closed fracture of the distal radius, unspecified
S234300	Closed fracture of ulna, styloid process
S234400	Closed fracture of ulna, lower epiphysis
S234500	Closed fracture distal ulna, unspecified
S234600	Closed fracture radius and ulna, distal
S234B00	Closed fracture radial styloid
S234C00	Closed fracture distal radius, intra-articular, die-punch
S234D00	Closed fracture distal radius, extra-articular, other type
S234E00	Closed fracture distal radius, intra-articular, other type
S234z00	Closed fracture of forearm, lower end, NOS
S23B.00	Fracture of lower end of radius
S242.00	Fracture at wrist and hand level
S23C.00	Fracture of lower end of both ulna and radius
S30..00	Fracture of neck of femur
S300.00	Closed fracture proximal femur, transcervical
S300000	Closed fracture proximal femur, intracapsular section, unspecified
S300100	Closed fracture proximal femur, transepiphyseal
S300200	Closed fracture proximal femur, midcervical section
S300300	Closed fracture proximal femur, basicervical
S300400	Closed fracture head of femur
S300500	Closed fracture proximal femur, subcapital, Garden grade unspecified
S300600	Closed fracture proximal femur, subcapital, Garden grade I
S300700	Closed fracture proximal femur, subcapital, Garden grade II
S300800	Closed fracture proximal femur, subcapital, Garden grade III
S300900	Closed fracture proximal femur, subcapital, Garden grade IV
S300A00	Closed fracture of femur, upper epiphysis
S300y00	Closed fracture proximal femur, other transcervical
S300z00	Closed fracture proximal femur, transcervical, not otherwise specified
S302.00	Closed fracture of proximal femur, pertrochanteric
S302000	Closed fracture of proximal femur, trochanteric section, unspecified
S302100	Closed fracture proximal femur, intertrochanteric, two part
S302200	Closed fracture proximal femur, subtrochanteric
S302300	Closed fracture proximal femur, intertrochanteric, comminuted
S302400	Closed fracture of femur, intertrochanteric
S302z00	Closed fracture of proximal femur, pertrochanteric section, not otherwise specified
S304.00	Pertrochanteric fracture
S305.00	Subtrochanteric fracture
S30w.00	Closed fracture of unspecified proximal femur
S30y.00	Closed fracture of neck of femur NOS
S31z.00	Fracture of femur, NOS
S4B0100	Closed fracture-dislocation superior radio-ulnar joint
S4C..00	Fracture-dislocation or subluxation of wrist
S4C0.00	Closed fracture dislocation of wrist
S4C0000	Closed fracture-dislocation distal radio-ulnar joint
S4C0100	Closed fracture-dislocation radiocarpal joint
S4C2.00	Closed fracture-subluxation of the wrist
S4C2000	Closed fracture-subluxation, distal radio-ulnar jt
S4C2100	Closed fracture-subluxation radiocarpal joint
S4E..00	Fracture-dislocation or subluxation hip
S4E0.00	Closed fracture-dislocation, hip joint
S4E2.00	Closed fracture-subluxation, hip joint
SC01.00	Late effect of fracture of spine and trunk without mention of cord lesion
SC01100	Late effect of fracture of thoracic vertebra
SC01200	Late effect of fracture of lumbar vertebra
SC3D400	Sequelae of fracture of femur
S31..00	Other fracture of femur
7K1Jd00	Closed reduction of intracapsular fracture of neck of femur and internal fixation using a dynamic hip screw
7K1Y000	Remanipulation of intracapsular fracture of neck of femur and fixation using nail or screw

6
