# Supplementary material for: Trends in incidence of recorded diagnosis of osteoporosis, osteopenia, and fragility fractures in people aged 50 years and above: retrospective cohort study using UK primary care data
Source: Osteoporos Int. 2023 May 10;34(8):1411–27. doi: 10.1007/s00198-023-06739-1 (PMC10382342; doi:10.1007/s00198-023-06739-1)
Supplement: Supplementary file 1 — Supplementary file1 (DOCX 54.9 KB) [file 198_2023_6739_MOESM1_ESM.docx]

**Supplementary Table S1. Crude and adjusted* incidence rates of hip fracture stratified by sex (2000-2018) (N=3,311,451) (Men N=1,583,550; Women N=1,727,901).**

| **Age (years)** | **Men – Crude IR per 10,000 PY (95%CI)** | **Women - Crude IR per 10,000 PY (95%CI)** | **Men – Adjusted* IRR (95%CI)** | **Women – Adjusted* IRR (95%CI)** |
| --- | --- | --- | --- | --- |
| All ages | 10.44 (10.26-10.63) | 27.30 (27.02-27.58) | 1 (Ref.) | 1.96 (1.92-2.00) |
| 50-54 | 1.89 (1.73-2.08) | 2.09 (1.91-2.28) | 1 (Ref.) | 1 (Ref.) |
| 55-59 | 2.53 (2.32-2.74) | 3.82 (3.57-4.08) | 1.34 (1.19-1.52) | 1.83 (1.64-2.04) |
| 60-64 | 3.75 (3.48-4.03) | 6.59 (6.24-6.95) | 2.00 (1.78-2.24) | 3.15 (2.84-3.49) |
| 65-69 | 5.79 (5.43-6.16) | 10.98 (10.50-11.48) | 3.07 (2.75-3.43) | 5.24 (4.75-5.78) |
| 70-74 | 9.87 (9.35-10.41) | 21.30 (20.58-22.05) | 5.26 (4.73-5.84) | 10.16 (9.25-11.17) |
| 75-79 | 19.54 (18.70-20.42) | 42.86 (41.73-44.01) | 10.43 (9.42-11.54) | 20.45 (18.65-22.42) |
| 80-84 | 37.24 (35.79-38.72) | 81.60 (79.82-83.41) | 19.86 (17.97-21.94) | 38.91 (35.54-42.60) |
| 85-89 | 69.55 (66.74-72.44) | 137.22 (134.27-140.22) | 36.90 (33.38-40.81) | 65.51 (59.83-71.72) |
| 90-99 | 113.93 (108.14-119.95) | 192.55 (187.80-197.38) | 60.16 (54.15-66.85) | 92.33 (84.26-101.17) |
| **Townsend quintile** | **Men – Crude IR per 10,000 PY (95%CI)** | **Women - Crude IR per 10,000 PY (95%CI)** | **Men – Adjusted* IRR (95%CI)** | **Women – Adjusted* IRR (95%CI)** |
| 1 (least deprived) | 8.30 (7.99-8.61) | 21.82 (21.34-22.31) | 1 (Ref.) | 1 (Ref.) |
| 2 | 9.68 (9.32-10.05) | 25.61 (25.05-26.17) | 1.09 (1.03-1.15) | 1.03 (0.99-1.06) |
| 3 | 10.73 (10.32-11.15) | 28.51 (27.89-29.15) | 1.22 (1.16-1.29) | 1.09 (1.05-1.13) |
| 4 | 12.14 (11.66-12.65) | 32.50 (31.75-33.26) | 1.37 (1.29-1.45) | 1.15 (1.11-1.19) |
| 5 (most deprived) | 14.75 (14.08-15.43) | 34.62 (33.65-35.61) | 1.70 (1.60-1.81) | 1.20 (1.15-1.25) |
| **Year** | **Men – Crude IR per 10,000 PY (95%CI)** | **Women - Crude IR per 10,000 PY (95%CI)** | **Men – Adjusted* IRR (95%CI)** | **Women – Adjusted* IRR (95%CI)** |
| 2000 | 8.54 (7.62-9.54) | 26.01 (24.48-27.60) | 1 (Ref.) | 1 (Ref.) |
| 2001 | 8.86 (8.00-9.79) | 25.33 (23.95-26.77) | 1.03 (0.88-1.19) | 0.97 (0.90-1.05) |
| 2002 | 8.51 (7.74-9.34) | 26.48 (25.19-27.83) | 0.99 (0.86-1.15) | 1.03 (0.95-1.11) |
| 2003 | 9.62 (8.84-10.45) | 27.22 (25.97-28.51) | 1.12 (0.98-1.29) | 1.06 (0.99-1.15) |
| 2004 | 9.11 (8.38-9.88) | 27.57 (26.37-28.81) | 1.07 (0.93-1.23) | 1.09 (1.01-1.17) |
| 2005 | 9.36 (8.65-10.12) | 27.65 (26.48-28.86) | 1.09 (0.95-1.25) | 1.09 (1.01-1.17) |
| 2006 | 9.15 (8.46-9.89) | 27.42 (26.28-28.61) | 1.05 (0.92-1.21) | 1.08 (1.00-1.16) |
| 2007 | 9.46 (8.77-10.20) | 27.51 (26.38-28.68) | 1.08 (0.94-1.23) | 1.08 (1.01-1.17) |
| 2008 | 9.72 (9.03-10.46) | 27.92 (26.79-29.08) | 1.10 (0.97-1.26) | 1.10 (1.03-1.19) |
| 2009 | 10.16 (9.45-10.90) | 27.03 (25.93-28.17) | 1.14 (1.00-1.31) | 1.07 (1.00-1.16) |
| 2010 | 11.59 (10.83-12.39) | 27.59 (26.47-28.75) | 1.29 (1.13-1.47) | 1.10 (1.02-1.18) |
| 2011 | 10.92 (10.19-11.69) | 28.01 (26.89-29.17) | 1.20 (1.05-1.37) | 1.11 (1.03-1.19) |
| 2012 | 10.80 (10.08-11.56) | 28.00 (26.88-29.15) | 1.17 (1.03-1.34) | 1.11 (1.03-1.20) |
| 2013 | 12.14 (11.37-12.96) | 28.19 (27.05-29.36) | 1.31 (1.15-1.50) | 1.13 (1.05-1.22) |
| 2014 | 11.97 (11.18-12.80) | 27.06 (25.92-28.24) | 1.28 (1.13-1.46) | 1.08 (1.01-1.17) |
| 2015 | 11.83 (10.99-12.72) | 26.78 (25.56-28.05) | 1.28 (1.12-1.46) | 1.09 (1.01-1.17) |
| 2016 | 12.57 (11.62-13.58) | 26.52 (25.18-27.91) | 1.37 (1.19-1.57) | 1.09 (1.01-1.18) |
| 2017 | 12.14 (11.13-13.21) | 28.37 (26.87-29.92) | 1.31 (1.14-1.51) | 1.16 (1.07-1.26) |
| 2018 | 11.09 (10.07-12.18) | 25.74 (24.24-27.32) | 1.18 (1.02-1.37) | 1.05 (0.96-1.14) |

*Adjusted for age, Townsend quintile of social deprivation, calendar year, and clustering by practice effect

**Supplementary Table S2. Crude and adjusted* incidence rates of vertebral fracture stratified by sex (2000-2018) (N=3,332,406) (Men N=1,588,834; Women N=1,743,572).**

| **Age (years)** | **Men - Crude IR per 10,000 PY (95%CI)** | **Women - Crude IR per 10,000 PY (95%CI)** | **Men – Adjusted* IRR (95%CI)** | **Women – Adjusted* IRR (95%CI)** |
| --- | --- | --- | --- | --- |
| All ages | 4.60 (4.47-4.72) | 9.47 (9.31-9.64) | 1 (Ref.) | 1.78 (1.73-1.84) |
| 50-54 | 1.99 (1.82-2.18) | 2.08 (1.90-2.27) | 1 (Ref.) | 1 (Ref.) |
| 55-59 | 2.15 (1.96-2.35) | 3.08 (2.86-3.32) | 1.09 (0.96-1.24) | 1.50 (1.34-1.69) |
| 60-64 | 2.93 (2.69-3.17) | 4.61 (4.32-4.92) | 1.48 (1.31-1.67) | 2.24 (2.01-2.49) |
| 65-69 | 3.91 (3.61-4.21) | 7.43 (7.04-7.84) | 1.95 (1.73-2.19) | 3.58 (3.23-3.97) |
| 70-74 | 6.01 (5.60-6.43) | 11.38 (10.85-11.93) | 3.04 (2.71-3.40) | 5.55 (5.03-6.14) |
| 75-79 | 8.40 (7.85-8.98) | 18.13 (17.40-18.88) | 4.29 (3.84-4.80) | 8.96 (8.13-9.88) |
| 80-84 | 11.69 (10.89-12.53) | 24.70 (23.73-25.69) | 5.95 (5.31-6.67) | 12.28 (11.15-13.53) |
| 85-89 | 15.78 (14.47-17.18) | 26.69 (25.43-27.99) | 7.96 (7.04-9.01) | 13.26 (12.00-14.67) |
| 90-99 | 18.30 (16.06-20.77) | 23.64 (22.07-25.29) | 9.16 (7.84-10.70) | 11.80 (10.55-13.18) |
| **Townsend quintile** | **Men - Crude IR per 10,000 PY (95%CI)** | **Women - Crude IR per 10,000 PY (95%CI)** | **Men – Adjusted* IRR (95%CI)** | **Women – Adjusted* IRR (95%CI)** |
| 1 (least deprived) | 3.72 (3.52-3.93) | 7.74 (7.46-8.04) | 1 (Ref.) | 1 (Ref.) |
| 2 | 4.54 (4.29-4.79) | 9.21 (8.88-9.54) | 1.11 (1.03-1.21) | 1.07 (1.01-1.13) |
| 3 | 4.74 (4.47-5.02) | 9.37 (9.02-9.74) | 1.20 (1.11-1.31) | 1.09 (1.03-1.15) |
| 4 | 5.18 (4.86-5.51) | 11.21 (10.77-11.66) | 1.30 (1.19-1.42) | 1.22 (1.15-1.29) |
| 5 (most deprived) | 5.89 (5.48-6.33) | 11.96 (11.40-12.55) | 1.46 (1.32-1.61) | 1.26 (1.18-1.35) |
| **Year** | **Men - Crude IR per 10,000 PY (95%CI)** | **Women - Crude IR per 10,000 PY (95%CI)** | **Men – Adjusted* IRR (95%CI)** | **Women – Adjusted* IRR (95%CI)** |
| 2000 | 2.54 (2.05-3.12) | 6.46 (5.72-7.28) | 1 (Ref.) | 1 (Ref.) |
| 2001 | 2.41 (1.97-2.92) | 6.46 (5.77-7.20) | 0.95 (0.72-1.26) | 1.01 (0.86-1.19) |
| 2002 | 2.93 (2.48-3.43) | 7.30 (6.63-8.02) | 1.15 (0.88-1.48) | 1.12 (0.96-1.31) |
| 2003 | 2.96 (2.54-3.44) | 7.10 (6.47-7.77) | 1.16 (0.90-1.49) | 1.09 (0.94-1.27) |
| 2004 | 3.81 (3.35-4.32) | 7.58 (6.96-8.24) | 1.50 (1.18-1.91) | 1.18 (1.02-1.37) |
| 2005 | 3.42 (3.00-3.89) | 7.40 (6.80-8.04) | 1.35 (1.06-1.72) | 1.15 (1.00-1.33) |
| 2006 | 3.53 (3.11-4.00) | 7.07 (6.50-7.68) | 1.39 (1.09-1.76) | 1.10 (0.95-1.28) |
| 2007 | 3.69 (3.26-4.16) | 8.15 (7.54-8.80) | 1.43 (1.13-1.81) | 1.26 (1.10-1.46) |
| 2008 | 3.98 (3.54-4.46) | 9.12 (8.48-9.79) | 1.54 (1.22-1.95) | 1.41 (1.22-1.62) |
| 2009 | 4.26 (3.81-4.76) | 9.42 (8.77-10.10) | 1.65 (1.31-2.08) | 1.45 (1.26-1.67) |
| 2010 | 4.33 (3.87-4.82) | 9.68 (9.02-10.38) | 1.66 (1.31-2.09) | 1.49 (1.29-1.71) |
| 2011 | 5.17 (4.67-5.70) | 10.24 (9.57-10.95) | 1.96 (1.56-2.46) | 1.56 (1.36-1.79) |
| 2012 | 5.02 (4.53-5.54) | 10.70 (10.02-11.42) | 1.89 (1.50-2.37) | 1.62 (1.42-1.86) |
| 2013 | 5.96 (5.42-6.53) | 11.37 (10.66-12.12) | 2.24 (1.78-2.80) | 1.72 (1.50-1.97) |
| 2014 | 5.97 (5.42-6.56) | 11.44 (10.71-12.21) | 2.22 (1.77-2.78) | 1.71 (1.49-1.96) |
| 2015 | 6.32 (5.71-6.98) | 12.26 (11.44-13.13) | 2.36 (1.88-2.97) | 1.82 (1.58-2.09) |
| 2016 | 7.09 (6.38-7.86) | 12.76 (11.84-13.73) | 2.63 (2.09-3.31) | 1.85 (1.61-2.14) |
| 2017 | 6.86 (6.10-7.67) | 12.44 (11.46-13.48) | 2.47 (1.95-3.13) | 1.75 (1.51-2.03) |
| 2018 | 7.25 (6.44-8.15) | 13.70 (12.61-14.86) | 2.55 (2.01-3.24) | 1.90 (1.64-2.20) |

*Adjusted for age, Townsend quintile of social deprivation, calendar year, and clustering by practice effect

**Supplementary Table S3. Crude and adjusted* incidence of other fragility fracture stratified by sex (2000-2018) (N=3,236,170) (Men N=1,554,925; Women N=1,681,245).**

| **Age (years)** | **Men - Crude IR per 10,000 PY (95%CI)** | **Women - Crude IR per 10,000 PY (95%CI)** | **Men – Adjusted* IRR (95%CI)** | **Women – Adjusted* IRR (95%CI)** |
| --- | --- | --- | --- | --- |
| All ages | 16.75 (16.52-16.99) | 58.73 (58.31-59.16) | 1 (Ref.) | 3.23 (3.18-3.28) |
| 50-54 | 11.01 (10.59-11.45) | 20.59 (20.01-21.18) | 1 (Ref.) | 1 (Ref.) |
| 55-59 | 11.60 (11.16-12.06) | 31.55 (30.81-32.30) | 1.07 (1.02-1.13) | 1.56 (1.51-1.62) |
| 60-64 | 12.91 (12.41-13.42) | 42.17 (41.26-43.09) | 1.19 (1.12-1.25) | 2.05 (1.98-2.13) |
| 65-69 | 14.90 (14.32-15.50) | 54.89 (53.78-56.01) | 1.35 (1.28-1.43) | 2.66 (2.57-2.76) |
| 70-74 | 16.91 (16.22-17.62) | 67.99 (66.65-69.36) | 1.56 (1.47-1.65) | 3.38 (3.26-3.50) |
| 75-79 | 23.90 (22.96-24.88) | 91.34 (89.62-93.08) | 2.21 (2.09-2.33) | 4.59 (4.44-4.75) |
| 80-84 | 32.71 (31.35-34.12) | 118.11 (115.88-120.38) | 3.01 (2.84-3.19) | 5.99 (5.78-6.19) |
| 85-89 | 50.37 (47.98-52.86) | 146.81 (143.63-150.04) | 4.58 (4.31-4.87) | 7.38 (7.12-7.65) |
| 90-99 | 66.28 (61.87-70.91) | 167.13 (162.57-171.79) | 5.96 (5.52-6.45) | 8.46 (8.13-8.80) |
| **Townsend quintile** | **Men - Crude IR per 10,000 PY (95%CI)** | **Women - Crude IR per 10,000 PY (95%CI)** | **Men – Adjusted* IRR (95%CI)** | **Women – Adjusted* IRR (95%CI)** |
| 1 (least deprived) | 14.31 (13.90-14.72) | 53.40 (52.63-54.18) | 1 (Ref.) | 1 (Ref.) |
| 2 | 15.65 (15.19-16.12) | 57.07 (56.23-57.93) | 1.06 (1.02-1.11) | 1.01 (0.99-1.03) |
| 3 | 16.93 (16.42-17.46) | 59.61 (58.68-60.55) | 1.16 (1.11-1.22) | 1.04 (1.01-1.06) |
| 4 | 19.00 (18.38-19.63) | 63.54 (62.46-64.62) | 1.30 (1.24-1.36) | 1.07 (1.05-1.10) |
| 5 (most deprived) | 22.08 (21.25-22.93) | 67.00 (65.61-68.41) | 1.46 (1.39-1.54) | 1.09 (1.06-1.12) |
| **Year** | **Men - Crude IR per 10,000 PY (95%CI)** | **Women - Crude IR per 10,000 PY (95%CI)** | **Men – Adjusted* IRR (95%CI)** | **Women – Adjusted* IRR (95%CI)** |
| 2000 | 11.07 (10.01-12.22) | 33.21 (31.46-35.03) | 1 (Ref.) | 1 (Ref.) |
| 2001 | 12.71 (11.67-13.82) | 35.86 (34.19-37.59) | 1.15 (1.01-1.31) | 1.07 (1.00-1.15) |
| 2002 | 11.79 (10.87-12.77) | 37.87 (36.29-39.49) | 1.07 (0.94-1.22) | 1.12 (1.05-1.20) |
| 2003 | 12.91 (12.00-13.88) | 38.69 (37.17-40.25) | 1.17 (1.04-1.32) | 1.14 (1.07-1.22) |
| 2004 | 12.72 (11.85-13.63) | 38.05 (36.62-39.53) | 1.16 (1.03-1.31) | 1.13 (1.06-1.21) |
| 2005 | 12.10 (11.28-12.97) | 41.26 (39.80-42.75) | 1.10 (0.98-1.24) | 1.22 (1.15-1.30) |
| 2006 | 11.86 (11.06-12.70) | 39.03 (37.64-40.47) | 1.08 (0.96-1.22) | 1.16 (1.09-1.24) |
| 2007 | 12.75 (11.93-13.60) | 40.47 (39.07-41.91) | 1.16 (1.03-1.30) | 1.20 (1.13-1.28) |
| 2008 | 12.42 (11.63-13.26) | 52.83 (51.24-54.46) | 1.12 (1.00-1.26) | 1.57 (1.47-1.67) |
| 2009 | 15.55 (14.67-16.48) | 81.93 (79.96-83.94) | 1.40 (1.25-1.57) | 2.45 (2.31-2.60) |
| 2010 | 16.62 (15.70-17.58) | 70.92 (69.07-72.81) | 1.49 (1.33-1.67) | 2.12 (2.00-2.25) |
| 2011 | 16.01 (15.11-16.95) | 67.28 (65.49-69.11) | 1.42 (1.27-1.60) | 2.01 (1.89-2.13) |
| 2012 | 25.04 (23.92-26.20) | 89.54 (87.48-91.64) | 2.21 (1.98-2.46) | 2.67 (2.51-2.83) |
| 2013 | 24.78 (23.65-25.95) | 83.27 (81.25-85.33) | 2.18 (1.95-2.43) | 2.48 (2.34-2.63) |
| 2014 | 25.44 (24.26-26.65) | 82.64 (80.57-84.74) | 2.21 (1.98-2.47) | 2.44 (2.30-2.59) |
| 2015 | 23.74 (22.52-25.01) | 77.95 (75.78-80.16) | 2.04 (1.82-2.28) | 2.27 (2.13-2.41) |
| 2016 | 19.37 (18.16-20.63) | 62.58 (60.44-64.76) | 1.67 (1.48-1.88) | 1.82 (1.71-1.95) |
| 2017 | 19.47 (18.17-20.84) | 58.62 (56.39-60.92) | 1.66 (1.47-1.88) | 1.68 (1.57-1.80) |
| 2018 | 17.72 (16.41-19.11) | 50.93 (48.73-53.20) | 1.50 (1.32-1.70) | 1.45 (1.35-1.56) |

*Adjusted for age, Townsend quintile of social deprivation, calendar year, and clustering by practice effect

**Supplementary Graph S1. Osteoporosis Incidence – Margins plot interaction age by sex.**

**Supplementary Graph S2. Osteopenia Incidence – Margins plot interaction age by sex.**

**Supplementary Graph S3. Fragility fracture Incidence – Margins plot interaction age by sex.**
